# Supplementary material for: The Dual Prey-Inactivation Strategy of Spiders—In-Depth Venomic Analysis of Cupiennius salei
Source: Toxins (Basel). 2019 Mar 19;11(3):167. doi: 10.3390/toxins11030167 (PMC6468893; doi:10.3390/toxins11030167)
Supplement: Supplementary file 1 [file toxins-11-00167-s001.zip › Supplementary Fig S1.1-S1.19/Supplementary Fig S1.4.pdf]

Supplementary Fig S1.4

CLUSTAL O(1.2.4) multiple sequence alignment: Venom serine proteases 2

```
VSP2_S1_CUPSA          MIAAICVL-CCLQLIRGIPLEQISEADTRPLCPCGDDDSSLNTRVVGGHAASRHQFPYAS
tr_A9QQB1_LYCSI        ------------------------------------------------------------
VSP2_ALOMA             MFAAICIL-WSLQLIQGSPLE-LSQEDTRPLCPCGDNGRPLNTRVVGGHAVPRHEFPFAS
VSP2_VIRFA             MIAAICLL-LYLQLIQGKPLERLYENDTRPLCPCGSNSSSVHTRVVGGQPVGRNVFPYAS
tr_A0A087T1C2_STEMI    MLPPIWFLFVFHQTVAELQINSLADSDTRPRCECGANRSSIKARVVGGHNAGKGIFPYAI


VSP2_S1_CUPSA          SILFRPHFGD--RELREFIRSTPFCGGTLITDRHVITAAHCLKERFPEDIAVDVGDYSLK
tr_A9QQB1_LYCSI        ------------------------------------------------------------
VSP2_ALOMA             NLLFRPFEEYYSGGRQASQKLSPFCGGTLITDRHILTAAHCLKDRDPEDLAVDVGDYSLR
VSP2_VIRFA             GLLVNSDYEE------RGVNSQPFCGGTLITDRHVLTAAHCLRDRIAHEVSVDVGDYSLR
tr_A0A087T1C2_STEMI    GLLDAEREDE----QSRIMRSQPFCGATLVSDRHVLTAAHCVKERTADHIAVDIGDYNLQ


VSP2_S1_CUPSA          EKRGKQILNSRNLTRFPEYIKTSFHTDIAIVELEHPVQFRSGLMTARLPPPDLDLKPGTT
tr_A9QQB1_LYCSI        ---------------------------IGVIELEHPVRFRSEMRTALLANQGLQLKPGTT
VSP2_ALOMA             EKKDRQVVNVRSLTKFPEYTRDSFHTDIGVIELEHPVRFRPGMRTALLPNQDLELKPGTT
VSP2_VIRFA             DESDGQVIGARNLTKYPEYTSGSFHTDIGIVELEKPVPFHLGIQTAPLPSPNLYLKPGTT
tr_A0A087T1C2_STEMI    EVREDNHHIVRNITKYPDYIPGKFHSDIAVIEFERPVDWSSGVRAAWLPSSSLELEPGTT
                                                  *.::*:*:** :   : :* *   .* *:****

VSP2_S1_CUPSA          VSVYGWGRLSYYGDHPDVLNSVDLPVVDSKQCQPKFVSRIEPAMICAGGQKGKDACIGDS
tr_A9QQB1_LYCSI        VSVYGWGRLSYFGGHPDVLNSVDLPVVRNEECQQNFVSRIEPSMICAGGLEGKDACIGDS
VSP2_ALOMA             VSVYGWGRLSYFGGHPDVLNSVDLPVVRNDLCQHEFVSRIEPSMICAGGQEGKDACIGDS
VSP2_VIRFA             VTVYGWGRLSYTGGHPDELHAVNLPVMDNDECRPKFVSKIERSMICAGGQKGKDACIGDS
tr_A0A087T1C2_STEMI    VSVYGWGRLKYGGSHPDILQTVELPVVNSTECQTEFVTVIEPSMLCAGGESGKDACIGDS
                       *:*******.* *.*** *::*:***: .  *: :**: ** :*:**** .*********

VSP2_S1_CUPSA          GSGLVVRLDNKFVLCGVVSFGRRCALPDVPGVYTRVSSFVDWILKQTQSASCRPCVYGKS
tr_A9QQB1_LYCSI        GSGLMVRLDNEFVLCGVVSFGRKCALPHVPGVYTKVSSYTDWILDQTRSASCRPCVYDK-
VSP2_ALOMA             GSGLMVRLDNEFVLCGVVSFGRKCALPHVPGVYTRVSSYTDWILDQTRSASCRPCVY---
VSP2_VIRFA             GSGLVVRLDNQFVLCGVVSFGRRCALPDIPGVYTRVSSYVDWVLQHTQTAECRPCIYGEN
tr_A0A087T1C2_STEMI    GSGLVVRLDNEYVLCGLVSFGRRCALPHVPGVYTRVSSFIDWIYNQTLSSDCKPCIYTEE
                       ****:*****::****:*****:****.:*****:***: **: .:* ::.*:**:*

VSP2_S1_CUPSA          ----------
tr_A9QQB1_LYCSI        ----------
VSP2_ALOMA             ----------
VSP2_VIRFA             ----------
tr_A0A087T1C2_STEMI    EESFRNIFKI
```

Percent Identity Matrix - created by Clustal2.1

1: VSP2_S1_CUPSA	100.00	77.63	73.04	67.58	56.95
2: tr_A9QQB1_LYCSI	77.63	100.00	92.67	69.08	67.76
3: VSP2_ALOMA	73.04	92.67	100.00	67.82	56.70
4: VSP2_VIRFA	67.58	69.08	67.82	100.00	55.97
5: tr_A0A087T1C2_STEMI	56.95	67.76	56.70	55.97	100.00

Proteomic coverage trypsin

>VSP2_S1_CUPSA venom serine protease 2

MIAAICVLCC LQLIRGIPLE QISEADTRPL CPCGDDDSSL NTRVVGGHAA SRHQFPYASS ILFRPHFGDR ELREFIRSTP
FCGGTLITDR HVITAAHCLK ERFPEDIAVD VGDYSLKEKR GKQILNSRNL TRFPEYIKTS FHTDIAIVEL EHPVQFRSGL
MTARLPPPDL DLKPGTTVSV YGWGRLSYYG DHPDVLNSVD LPVVDSKQCQ PKFVSRIEPA MICAGGQKGK DACIGDSGSG
LVVRLDNKFV LCGVVSFGRR CALPDVPGVY TRVSSFVDWI LKQTQSASCR PCVYGKS

Proteomic coverage chymotrypsin

>VSP2_S1_CUPSA venom serine protease 2

MIAAICVLCC LQLIRG**IPLE QISEADTRPL** CPCGDDDSSL NTR**VVGGHAA SRHQFPYASS ILFRPHFGDR ELREF**IRSTP F**CGGTLITDR HVITAAHCLK ERFPEDIAVD VGDY**SLKEKR GKQILNSRNL TRFPEYIKT**S FHTDIAIVEL EHPVQF**RSGL **MTARLPPPDL DLKPGTTVSV Y**GWGRL**SYYG DHPDVLNSVD LPVVDSKQCQ PKFVS**R**IEPA MICAGGQKGK DACIGDSGSG LVVRLDNKF**V LCGVVSF**GRR CALPDVPGVY T**RVSSFVDW**I LKQTQSASCR PCVYGKS**

Supplementary Fig S1.4 Multiple sequence alignment of VSP2 from *C. salei* (VSP2_S1_CUPSA [MH766623]), *L. singoriensis* (tr_A9QQB1_LYCSI) *A. marikovskyi* (VSP2_ALOMA), *V. fasciatus* (VSP2_VIRFA), and *S. mimosarum* (tr_A0A087T1C2_STEMI). Signal peptides are marked in black characters, cysteines in white with a black box, positively charged amino acids in pink, negatively charged amino acids in blue, hydrophobic amino acids in red and polar amino acids in green. The catalytic triad is highlighted in gray, the involved amino acid residues of the proposed active site binding structure are highlighted in yellow. A possible processing site to obtain the proposed activated heterodimeric serine protease is highlighted in red.
